# Retraction Note: Yes-associated protein (YAP) binds to HIF-1α and sustains HIF-1α protein stability to promote hepatocellular carcinoma cell glycolysis under hypoxic stress

**DOI:** 10.1186/s13046-026-03728-0

**Published:** 2026-05-14

**Authors:** Xiaodong Zhang, Yan Li, Yingbo Ma, Liang Yang, Tao Wang, Xin Meng, Zhihong Zong, Xun Sun, Xiangdong Hua, Hangyu Li

**Affiliations:** 1 Department of General Surgery, The Fourth Affiliated Hospital of China Medical University, 4 Chongshan East Street, Shenyang, 110032 Liaoning People’s Republic of China; 2 Department of Oncology, Tumour Angiogenesis and Microenvironment Laboratory (TAML), The First Affiliated Hospital of Jinzhou Medical College, Jinzhou, China; 3 Department of Pathology, The Shengjing Hospital of China Medical University, Shenyang, Liaoning China; 4 Department of Biochemistry and Molecular Biology, College of Basic Medicine, China Medical University, Shenyang, China; 5 Department of Immunology, College of Basic Medicine, China Medical University, Shenyang, China; 6 Department of Hepatobiliary Surgery, Liaoning Cancer Hospital and Institute, Shenyang, China

**Retraction Note: J Exp Clin Cancer Res 37**,** 216 (2018)**

**https://doi.org/10.1186/s13046-018-0892-2**

The Editor-in-Chief has retracted this article. Three panels in Fig. 4H appear to partially overlap with rotation, despite showing different conditions. The alternative images provided by the authors were insufficient for a correction, and the authors did not provide a sufficient response to the concerns raised. The Editor has lost confidence in the data and conclusions of this article.

Author Hangyu Li agrees with this retraction. All other authors have not responded to correspondence from the publisher regarding this retraction.

